# Quarantine Conference at New Orleans

**Published:** 1899-02

**Authors:** 


### Abstracts and Selections.

### Quarantine Conference at New Orleans.—Texas was not Represented.

*From Galveston News.*

"By an almost unanimous vote the Conference of Boards of Health Representatives and Health Officers of three Southern States, held at New Orleans February 9th, adopted the amended Atlanta quarantine regulations for the good of the country in times of yellow fever scares and where yellow fever actually exists. But Texas is not a party to the agreement, and while the conference is expected to result in great good for the whole South, not only from a sanitary and scientific point of view, but commercially and in many other ways, Texas is destined to move along in the same old rut, with the same dangers threatening her as were experienced in past summers, when frights of yellow fever created a quarantine against this city and nearly the whole State. Texas has no State Board of Health, and Dr. Blunt, State Health Officer, was not present at the conference, nor was he represented as the head of the Health Department of this State by any of the several representatives from Texas who attended the conference."

This statement was obtained from Dr. J. F. Y. Paine, who, with Alderman J. D. Skinner, represented Galveston at the meeting.

Dr. Paine and Mr. Skinner returned home yesterday morning from the Crescent City. They took an active part in the conference, and Dr. Paine was identified in framing the amended resolutions and other measures adopted by the convention. Dr. Paine was chairman of the committee which framed the resolutions adopted as a substitute for three resolutions offered by President Souchon, of the Louisiana Board of Health. He said: "The conference was well attended and the business of the meeting was conducted with neatness and dispatch and was very harmonious. The Atlanta regulations were revised, amended and framed in a concise and clear condensation, so that they could be carried out to the letter. The original regulations were all right in themselves, but they were not practicable, and were too voluminous to admit of ready interpretation and practical observation. In other words the conference was a success, so far as accomplishing the purposes for which it was assembled, but Texas is not a party to the new regulations, and in so far as this State is concerned, there can be no good results obtained unless a change be made in the management of affairs of the State Health Department."

Dr. Blunt, State Health Officer, is quoted as having declared himself last summer to the effect that he would not be governed by the Atlanta regulations, and left the inference that he would not be a party to any agreement by any other conference in matters of quarantine. The authority for this statement is Dr. W. C. Fisher, City Health Officer of Galveston, who repeated the statement yesterday to a *News* reporter in the presence of Dr. Paine.

Drs. Fisher and Paine are both strong advocates of a State Board of Health, an institution of which Texas does not boast, and are greatly opposed to the system of controlling the State Health Department, as they expressed it yesterday at a discussion of quarantine matters.

The resolution passed at the New Orleans conference reads as follows:

"No locality shall be quarantined on account of cases of contagious diseases reported as suspicious or doubtful or disputed, provided the cases are properly isolated, the premises disinfected, the inmates and suspects thoroughly disinfected, and both inmates and suspects kept under proper observation by the local health authorities, all under the supervision and control of the State Board of Health, State Health Officer or United States Marine Hospital Service, and such suspected case or cases shall be reported to the health officers or Boards of Health of adjacent States, and they be invited to send representatives to view said case or cases; this resolution also to apply to sanitariums, hospitals and barracks."

City Health Officer was present yesterday when a *News* reporter called upon Dr. Paine to learn what had been done at the conference at New Orleans for the good of Galveston and all Texas. Dr. Paine is highly pleased with what was done at the conference, and believes that the results will be great when the regulations are applied. But he deplores the fact that Texas is not in the new arrangements, and that no benefits can be expected to accrue to this State under the present operations of the State Health Office.

Referring to the resolutions adopted at the conference, and also to the revision of the Atlanta regulations, Dr. Paine said that he believed the resolutions adopted will be productive of great good if adhered to. "But they will absolutely amount to nothing, so far as Texas is concerned, if Dr. Blunt is permitted to use his authority as arbitrarily in the management of the State Health Office as he has done in the past," continued Dr. Paine. He said that Texas was represented by the municipal boards of health and individuals from all parts of South Texas, but the State Health Department, as such, was not represented, and that Dr. Blunt had gone on record as opposing the Atlanta regulations.

It was here that Dr. Fisher made the statement about Dr. Blunt saying that he would not abide by the Atlanta regulations, and added that Dr. Blunt, on the same occasion, had stated that he "attributed the outbreak of yellow fever last summer in Mississippi and Louisiana to the carrying out of the Atlanta regulations in these two States."

Dr. Paine said that the new arrangements were the best ever made in reaching the relief sought after in times of yellow fever scares. Under the new regulations, where suspicious cases are reported, the city or State will not be quarantined on mere "suspected" cases, nor will the commerce or trafic be blocked by the quarantine regulations on the mere reporting of a "suspect." But Texas cannot share in the benefits, he said, because State Health Officer Blunt will not comply with this agreement, and, of course, the other States will not permit of any traffic or travel from Texas in times of danger.

"Galveston is a great and growing port, and Texas is a great and growing State, but we have not advanced one step in the right direction in fighting this great danger of quarantine," said Dr. Paine. "It is the same as fifty years ago, and the county and municipal boards of health are powerless to do anything as long as the State Health Officer opposes the movement. Under the present agreement we would not have experienced the quarantine which we did last year when the suspicious case was discovered at Fort Point. Dr. Fisher reported the case, isolated the soldier patient and all those who had come in contact with him, burned the quarters, and threw up every safeguard necessary. Yet a quarantine was established against this city and port, and just such instances as that might keep all Texas tied up all summer if permitted to exist. The new rules provide for such emergencies, and while the suspect is removed to a safe place and all necessary precautions taken, the whole State, or even town, is not made to suffer for an indefinite period to learn in the end that the case is not yellow fever. I stated at the conference that there is no man living who can positively diagnose a case of yellow fever with the combination of symptoms appearing in the early stages of the contagion. Even the Marine Hospital Service is not composed of men immaculate and infallible. If a city, town or State is going to be shut out from the world every time a suspicious case appears, it is high time the regulations were changed."

AN EXCELLENT MEETING.

Colonel Skinner returned from New Orleans with Dr. Paine. "It was an excellent meeting, made of representative men from all the States affected by the quarantine matters. The rules which we formulated and which the different States will be asked to ratify, I think, will do away with all the difficulties that have presented themselves during quarantine periods in the past. These rules look to the moving of merchandise and mails when there are cases of sickness of a suspicious nature. It is required that there shall be no absolute quarantine so long as there is but one case of suspicion, or even several cases where the patients are isolated, together with those who have come in contact with them.

"Texas had the largest delegation of any State, and I think it was a representation that compared very favorably with any of them. There were several leading doctors from Houston, and Dr. Harrison, of Columbus, was there. The Galveston delegation was opposed to the Caffery bill, though those in New Orleans favored it. While the New Orleans people contend to the contrary, we believe that it means that the quarantine matter will be placed in the hands of the Marine Hospital Service, and that is what we do not want to see. The New Orleans people wired the Galveston Cotton Exchange asking them to endorse the Caffery bill, and we wired asking them to leave the matter in the hands of the representatives at the meeting. This they did, and we expressed our opposition to the bill."

REVISED AND AMENDED RULES ADOPTED AT THE NEW ORLEANS MEETING.

Page 6, No. 27, shall read: Parties coming from localities infected with yellow fever should not be allowed to enter quarantining localities capable of being infected by yellow fever, unless they have had their persons, clothing, baggage, etc., disinfected as needed, and unless they have remained at the station ten days after such thorough disinfection, and places holding communication with localities under insufficient restrictions may themselves be held in quarantine.

Page 7, No. 33, shall read: Persons arriving at the disinfection stations will have their clothes and effects disinfected.

Page 7, No. 36, shall be stricken out. It reads as follows:

36. Articles not amenable to this treatment will be immersed in a solution of 1 to 1000 of bichloride of mercury for one hour; or they will be placed in the formaldehyde chamber fed from a generator for one hour, drying them first and spreading them in the chamber.

Page 7, after No. 46, add No. 46b. All articles shall be new, clean and dry.

Add No. 46c. Special conditions of infection.

1. When fever exists in a sporadic form.

Merchandise under the above conditions can be shipped.

2. Whenever it is more than sporadic, but not general.

Merchandise of the above character may be shipped from the wholesale district of a city, except such as from its liability to infection would be especially apt to conserve it, such as fruit, vegetables in open crates, straw, sawdust, excelsior and similar articles used for packing.

These articles can be shipped only if they have been preserved from possible exposure to infection, or have been disinfected.

Page 7, No. 47, shall read as follows:

CLASS I.

The following articles should be admitted without disinfection or restrictions of any sort:

(a) All new and dry material, unpacked, such as lumber, machinery, brick, tiling, bar and sheet iron, tin, steel, agricultural implements—no part of which is textile; iron ties, stoves, saddlery, not upholstered; rubber belting, rubber hose, linoleum, wagons, new trunks, hardware without packing, lime, ice and salt in bulk, turpentine, rosin, stone, gravel, coal, coke, cement, grain in carloads, cooperage, oysters and fish packed in ice properly refrigerated.

(b) Original packages in clean and smooth wooden or metallic containers not broken or packed in an infected locality.

(c) Articles in such containers, put up and handled exclusively in the wholesale district, which from their nature or mode of packing are incapable of carrying infection, such as roasted coffee, refined sugar, coal oil, creosote, acids, beans, peas, rice, salt meats and articles of a similar character.

(d) Fruits, sound, and taken directly in good condition from clean vessels or cars and transferred at wharves and railroad depots, not infected and in good sanitary condition, immediately to the disinfected cars or vessels for shipment, provided the vessel has complied with all quarantine regulations.

(e) Fruit, vegetables and western produce in barrels or boxes directly transferred as above.

(f) Freight in good sanitary condition, taken directly from clean vessels or cars to cars or vessels at a wharf or railroad siding, not infected and in good sanitary condition.

(g) Livestock and poultry.

Page 7, Nos. 48, 49, 50 and 51, shall be stricken out. These read as follows:

48. Fruits, sound, and taken directly in good condition from clean vessels, and transferred at wharves not infected and in good sanitary condition immediately to the disinfected cars for shipment, require no disinfection.

49. Freight taken directly from clean vessels to cars at a wharf not infected, and in good sanitary condition, will require no disinfection.

50. Livestock and poultry are included in this list.

51. All disinfected cars to be placarded and way bills certified to by proper sanitary officers.

Page 8, No. 52, shall read as follows:

CLASS II.

The following articles will require only superficial disinfection, i. e., outside of containers:

(a) All goods in original wooden or metallic packages, not broken or packed in any infected locality, when not included in class 1, such as boots and shoes, dry goods, leather goods, drugs and chemicals, patent medicines, oiled and rubber clothing, sugar, canned fruits, canned vegetables, canned meats, canned oysters, canned fish, condensed milk, stoneware, tinware, tobacco, cigars, snuff, wines, tonics, liquors, cheese, flour, meal, grits, wooden ware, butter, tea, candles, soap, lard, starch, axle grease, iron roofing, saddle trees, raisins, matches, salted fish, molasses, rice, coffee, beans and peas in barrels, nuts, dried fruits, pickles, vinegar, olive oil, sauces, baking powder, soda, preserves.

(b) Articles which from their nature and mode of packing are incapable of receiving infection, and which sterilize the inside of the container, such as roasted coffee, refined sugar, molasses, coal oil, creosote, acids, and articles of similar character, when not included in class 1.

(c) Goods in textile material, not broken or packed in an infected locality and kept perfectly dry. This includes coffee, grain and spices in sacks; cured hams in canvass, Osnaburgs and other cotton goods in solid bales with close covering.

(d) Chemicals, patent medicines, drugs and druggists' sundries, not put up in an infected locality, when inclosed in glass, wood or metal; also hardware, when these articles are packed with sterilized excelsior.

Page 8, No. 53, shall be stricken out.

Page 8, No. 54, shall read as follows:

CLASS III.

Articles not in classes 1 and 2 may be shipped after disinfection. This refers to all classes of merchandise not in classes 1 and 2, and which are kept in stock for distribution at wholesale stores not exposed to any recognized infection. Articles that can be packed in excelsior in crates so as to render the excelsior and contents capable of disinfection belong to this class. Methods of disinfection are treated in a separate section of these regulations.

Page 8, Nos. 55, 56, 57 shall be stricken out. These read as follows:

55. When it has been exposed to infection, then it should be fumigated to be made harmless.

56. When desired, all goods of this character can be made to undergo a treatment of fumigation and disinfection before being certified to by the proper health authorities.

57. Articles mentioned above, that can be packed in excelsior, in perforated container, rendering the excelsior and contents capable of disinfection, will be disinfected and passed.

Page 8, No. 58, shall read:

CLASS IV.

No bedding or household effects shall be received for shipment under any conditions.

Page 8, after No. 59, add to No. 59b: Inspectors shall not certify to any of the above classes if not satisfied that the foregoing conditions have been complied with.

Page 8, No. 60, shall read: Regulations governing the repacking and disinfection of goods taken from original packages.

Page 8, Nos. 61 to 74, both inclusive, shall be stricken out and replaced by the following:

(a) Each establishment packing or repacking will

PROVIDE DISINFECTING CHAMBER

under the supervision of an inspector.

(b) The workmen, on arrival, will disinfect their hands and faces. They will then change their outer clothing for sterilized clothing, and remain in the work room during working hours.

(c) The workrooms and all the premises shall be kept clean.

(d) The outer clothing worn by the workmen during working hours shall be disinfected daily.

(e) The same precautions are required of the inspectors.

(f) If the goods to be packed are taken from previously opened packages, they must be disinfected.

(g) Work of this kind shall be done only in the wholesale districts.

Page 9, No. 76, shall read: Regulations governing workmen in factories:

Page 9, No. 77, shall read: The same regulations referring to workmen and premises of wholesale stores shall apply to factories.

Page 9, No. 78, shall read: In addition the goods manufactured, if liable to convey infection, must be disinfected.

Page 9, No. 85, after the last line add as a subheading:

85b. Regulations governing railroad traffic from an infected town to points south.

85c. Regulations governing freight traffic from an infected town to points south, as per 45, and following.

Page 10, No. 93, first line shall read: If not disinfected they shall be sent with windows, etc.

Page 10, after No. 93, add:

No. 93b. All disinfected cars must be placarded and way bills certified to by proper sanitary officers.

Page 10, No. 95, shall read: Letter mail needs no disinfection except in a marked epidemic.

Page 10, No. 97, after last line add as a subheading:

97b. Passenger traffic as per No. 27 and following.

Page 10, No. 98. Put in cut from Mississippi regulations.

Page 11, No. 123, shall read: This traffic should be, etc.

Page 11, No. 136, second line shall read: May be depopulated, etc.

Page 12, No. 141, shall read: If the inspection of a town in which yellow fever exists shows that all foci of infection, possible fomities and persons liable to develop the disease are under observation, the town should not be quarantined.

Page 12, No. 142, shall be stricken out. It reads: "Should either of these conditions fail, the town shall be quarantined.

Page 12, after No. 162, add No. 162b. The evidence of immunity shall be satisfactory to the health officer of the place to which he is bound.

Page 9, add to 75:

75b. Provided that this method meets with the approval of the various State Boards of Health or health officers where these goods are to be shipped.

Page 11. Strike out Nos. 132 and 134. These refer to the steamboats and read as follows: They may be carried on—132, by relays like railroad trains; 134, it is confessedly difficult but not impossible.

Add: That all municipal corporations and communities which are exposed to yellow fever infection in the Atlantic and gulf States

PROVIDE ISOLATION QUARTERS

for persons who may become infected or who may have been exposed to infection.

The committee also recommended a vote of thanks, which was given, to Dr. H. R. Carter, United States Marine Hospital Service, for his able exposition of quarantine principles, and urged that they be given the widest publicity. This letter of Dr. Carter's reads as follows:

NEW ORLEANS, LA., January 24, 1899.

*Dr. Edmond Souchon, President Louisiana Board of Health, New Orleans, La.*

DEAR DOCTORS—I am very sorry that I will not be able to attend the conference on February 9, as I am called off and leave today.

Inclosed please find a little paper I had hoped to present of "General Principles," on which quarantine measures, I think, ought to depend, and in proportion as they are founded on these principles they have, in my experience, been efficient and non-obstructive.

I think they will be found to have a bearing on the work which the convention is likely to consider, and thus have more than theoretical interest.

If you think it will be of any service to the convention, I beg that you will have it presented as written, either by the representative of the service, if he desires it, or yourself.

Very sincerely yours,

H. R. Carter,

Surgeon, Marine Hospital Service.

### DR. CARTER'S PAPER ON GENERAL PRINCIPLES.

A. *Purpose of Quarantine.*—1. The purpose of quarantine restrictions is to prevent the introduction of infectious or contagious diseases.

2. They should be sufficient for this purpose, and none save such as are necessary for this purpose should be imposed.

3. In case of doubt, the doubt should be thrown to the side of safety rather than of risk; but, in deciding on any measure, a balance should also be preserved between the risk which is obviated by its adoption and the loss which the measure entails.

4. Measures which, although safe in theory, yet are so difficult of execution that there is serious doubt that they will be carried out efficiently, cannot be depended on, and privileges depending on restrictions of this kind should not be allowed.

B. *Establishment of Foci of Yellow Fever.*—A focus of infection can be established only in an infectable place. To places in which such foci cannot be established, whether from location (latitude, altitude or other conditions), time of year (after frost), or other causes, yellow fever is not an infectious disease. Such places need not quarantine.

The same result may be obtained, i. e., not establishing a focus, by antiseptic treatment, of cases of yellow fever.

It is not generally to be depended on save in hospitals or tents.

C. *Conveyance of Yellow Fever.*—Yellow fever is usually conveyed from infected places by persons and personal effects, the latter already infected and the former having the fever in the stage of incubation. The former is by far the most common medium of conveyance.

This implies that the person or things have been exposed to infection.

Other things besides personal effects, such as articles of merchandise, may, of course, convey infection, but in point of fact seldom do.

The risk from persons depends on three factors:

1. That they have deen exposed to infection.
2. That they are susceptible to infection, if exposed.
3. That the period of incubation of the disease has not passed since last exposure.

If any one of these factors are lacking no risk can be conveyed by the person.

The risk from the effects of persons depends on:

1. Whether they have been exposed to infection.
2. Whether they have retained the infection to which they have been exposed—i. e., they have not been disinfected chemically or by aeration.

Persons and personal effects should be considered together.

Merchandise, other than personal effects, shipped from a place in which foci of infection of yellow fever exist, is dangerous in proportion to a combination of three factors:

1. Its exposure to infection.
2. Its inability to receive and convey it.
3. The measures adopted to free it from infection, if exposed to it.

Both of the first two factors must exist to render the merchandise dangerous in the first place, and even then it may be freed from danger by proper measures to free it from infection.

The first depends on:

(a) The degree of infection in the place.

(b) The place of storage and handling of the merchandise.

Until the infection of a city becomes general, the risk of infection is confined to the residences and places contiguous to them, and in the business portion of a city is rare, the wholesale business houses being practically free from it.

Reference is here had only to a city in which there is such a difference in residence and business portion.

Goods from the wholesale portion of such a town, unless the infection of the place be very general, are little apt to be exposed to infection.

Should the infection become very general, the wholesale district may be invaded.

The second depends on the nature of the surface of the merchandise. Smooth, clean, dry, non-absorbing surfaces will scarcely, even if exposed to infection, convey it.

The third, on the process of disinfection to which the merchandise has been subjected. This requires no explanation.

D. *Risk of Conveyance.*—The risk of communication with a place in which foci of yellow fever infection exist, is, among other things, dependent on and proportionate to:

1. Degree of infection.

Where the degree is small, the infection is, in general, confined to a small proportion of the residences, and the risk of conveying infection is then confined to persons and things which have been in this quarter.

2. Measures taken in and adjacent to this place.

If none of the persons exposed to infection and no infected articles be allowed to leave, there is no risk, and in proportion as this is done, the risk diminishes.

The nature of the quarantine restrictions, i. e., the nature of the communication allowed, should be modified by the risk, and thus depends partly on the above conditions.

